# Biophysical characterisation of the recombinant human frataxin precursor

**DOI:** 10.1002/2211-5463.12376

**Published:** 2018-01-25

**Authors:** Ignacio Hugo Castro, Alejandro Ferrari, María Georgina Herrera, Martín Ezequiel Noguera, Lorenzo Maso, Monica Benini, Alessandra Rufini, Roberto Testi, Paola Costantini, Javier Santos

**Affiliations:** ^1^ Institute of Biological Chemistry and Physicochemistry Dr Alejandro Paladini (UBA‐CONICET) University of Buenos Aires Argentina; ^2^ Department of Biology University of Padova Italy; ^3^ Laboratory of Signal Transduction Department of Biomedicine and Prevention University of Rome ‘‘Tor Vergata’’ Italy; ^4^ Fratagene Therapeutics srl Rome Italy

**Keywords:** aggregation, conformation, Friedreich's ataxia, precursor, stability, unfolding

## Abstract

Friedreich's ataxia is a disease caused by a decrease in the levels of expression or loss of functionality of the mitochondrial protein frataxin (FXN). The development of an active and stable recombinant variant of FXN is important for protein replacement therapy. Although valuable data about the mature form FXN81‐210 has been collected, not enough information is available about the conformation of the frataxin precursor (FXN1‐210). We investigated the conformation, stability and function of a recombinant precursor variant (His6‐TAT‐FXN1‐210), which includes a TAT peptide in the N‐terminal region to assist with transport across cell membranes. His6‐TAT‐FXN1‐210 was expressed in *Escherichia coli* and conditions were found for purifying folded protein free of aggregation, oxidation or degradation, even after freezing and thawing. The protein was found to be stable and monomeric, with the N‐terminal stretch (residues 1–89) mostly unstructured and the C‐terminal domain properly folded. The experimental data suggest a complex picture for the folding process of full‐length frataxin *in vitro*: the presence of the N‐terminal region increased the tendency of FXN to aggregate at high temperatures but this could be avoided by the addition of low concentrations of GdmCl. The purified precursor was translocated through cell membranes. In addition, immune response against His6‐TAT‐FXN1‐210 was measured, suggesting that the C‐terminal fragment was not immunogenic at the assayed protein concentrations. Finally, the recognition of recombinant FXN by cellular proteins was studied to evaluate its functionality. In this regard, cysteine desulfurase NFS1/ISD11/ISCU was activated *in vitro* by His6‐TAT‐FXN1‐210. Moreover, the results showed that His6‐TAT‐FXN1‐210 can be ubiquitinated *in vitro* by the recently identified frataxin E3 ligase RNF126, in a similar way as the FXN1‐210, suggesting that the His6‐TAT extension does not interfere with the ubiquitination machinery.

**Abbreviations**
ANS: 8‐anilino‐1‐naphthalene sulfonic acid
CD: circular dichroism
CFM: confocal fluorescence microscopy
CTR: C‐terminal region
FITC: fluorescein isothiocyanate
FRDA: Friedreich's ataxia
FXN: frataxin
GdmCl: guanidinium chloride
His6‐TAT‐FXN1‐210: the precursor of FXN including an N‐terminal His tag and a cell‐penetrating peptide
TAT: trans‐activator of transcription from HIV‐1

Friedreich's ataxia (FRDA) is a disease caused in 95% of the cases by a decrease in the levels of expression of frataxin (FXN) because of a GAA‐repeat expansion present within the first intron of both *fxn* alleles 1. In the remaining 5% of the FRDA patients, the loss of FXN functionality is caused by a combination of the typical expansion present in only one allele and a mutation in the other 2. The outcome of the lack of FXN functionality is an inefficient iron‐sulfur cluster assembly, with widespread enzymatic deficit and oxidative damage catalysed by an excess of labile iron 3, 4.

In humans, FXN is translated in the cytoplasm as a precursor of 210 residues (FXN1‐210). Then, this protein is imported into the mitochondrial matrix and processed in a two‐step process 5, 6. The first step yields the intermediate form (FXN42‐210), whereby the signal peptide for mitochondria import is excised. The second step of proteolysis results in the mature form (FXN81‐210). This form is functional as an activator of the NFS1/ISD11/ISCU desulfurase protein complex, which is involved in the mitochondrial iron‐sulfur cluster assembly 7, whereas several FXN mutants exhibited compromised activation 7, 8, 9. In addition, FXN was first described as an iron chaperone given its iron binding activity and it is related to redox balance inside the organelle 10, 11.

Significant efforts are being done by the scientific community to increase the concentration of active FXN inside mitochondria. One outstanding strategy involves the production of recombinant variants of FXN that have the capability of crossing the cell membrane and being imported into the mitochondrial matrix, thus yielding an active and stable form of the protein. Recently, to achieve this objective, a TAT‐derived peptide (TAT stands for trans‐activator of transcription from HIV‐1) was fused with FXN precursor 12, 13, 14, 15. TAT was one of the first cell‐penetrating proteins (CPPs) to be discovered. 16. Like other CPPs, HIV‐TAT (herein, just TAT) behaves as a transduction motif allowing cell penetration by crossing the cytoplasmic membrane. The amino acid sequence of TAT peptide is *RKKRRQRRR*. This peptide carries eight positive charges that are important for an efficient translocation. TAT peptide promotes the cellular uptake of coupled peptides and proteins, as well as oligonucleotides, siRNAs, nanoparticles and cell‐impermeable drug molecules 17.

Even though the precise mechanism of the TAT‐mediated translocation is unknown, some aspects of the process have been characterised over the last years. The TAT sequence is very rich in arginine and lysine residues, giving a nonamphipathic short moiety with a high positive charge, which favourably interacts with the phosphate groups on both sides of the cell membrane 18. Most likely, the mechanism of TAT translocation involves lipid perturbation and local membrane thinning with a pore formation 19. However, there are still some controversies about the TAT internalisation pathway.

For an eventual TAT‐FXN‐based therapy, protein conformation is one of the most important issues; but in the case of FXN, there is not enough information about the conformation of the precursor FXN1‐210. Nonetheless, there have been some reports concerning this issue, where it was described that the intermediate form (FXN42‐210) and variant FXN56‐210 exhibit a tendency to form soluble oligomers, or even, to aggregate in an irreversible fashion, yielding insoluble protein 20, 21. Furthermore, it has been observed that the N‐terminal stretch of these variants is most likely unfolded or at least disordered 22, 23. Moreover, the N‐terminal of FXN56‐210 is directly involved in the oligomerisation process 24. Thus, it is not obvious that the precursor will be well‐folded under *in vitro* conditions.

In this paper, we investigated the conformation and stability of a recombinant precursor variant (His6‐TAT‐FXN1‐210) that included a TAT peptide in the N‐terminal region. This allowed protein translocation across the cytoplasmic membrane. In addition, a histidine tag was incorporated for easy purification. Given that the protein exhibited a certain tendency to aggregate during freeze and thaw, we optimised buffer conditions to preserve the protein in a monomeric and active folded state.

## Results

### FXN precursor purification, optimisation of buffer conditions and conformational analysis

The His6‐TAT‐FXN1‐210 variant was produced in *E. coli* and expression conditions were optimised to purify the precursor variant from the soluble fraction. Even though the protein was produced using codon plus ROSETTA2pLysDE3 BL21(DE3) cells, the level of protein expression was substantially lower than the level observed for the mature form FXN90‐210 (data not shown). In addition, a significant fraction of protein was found in the insoluble fraction. Degradation of the full‐length FXN into a shorter species was observed consistently when cells were disrupted by sonication. By contrast, when bacteria were disrupted by a French press, the species of lower molecular weight was not observed. The latter strategy, therefore, was usually adopted, while purification was performed immediately after cell lysis.

Aggregation of the purified protein was observed during freeze and thaw, or even when the protein was maintained at 4 °C for 2 days. After testing various buffer conditions, we found that buffer containing 20 mm Tris‐HCl (pH 7.5), 300 mm NaCl, 1 mm EDTA, 1 mm DTT, 15% glycerol to be appropriate as this amount of glycerol was enough to preserve the protein in a soluble, non‐aggregated form at 4 °C and even after freezing and thawing.

It is worth noting that the presence of EDTA and DTT was critical for inhibiting the oxidation and degradation of the protein in the early stages of the purification process. The FXN precursor has two cysteine residues in the N‐terminal stretch (Cys 37 and Cys 50, GenBank: AAH48097.1) that might confer propensity to oxidation by forming intra‐ and interdisulfide bonds. In fact, after long incubation times in the absence of DTT, no thiols were detected (data not shown); this result suggests oxidation. On the other hand, it is well known that FXN binds iron and that the precursor may undergo autoproteolysis mediated by the metal ion, yielding the shorter form FXN78‐210, as detected by SDS/PAGE and ESI‐MS 25.

**Figure 1:**
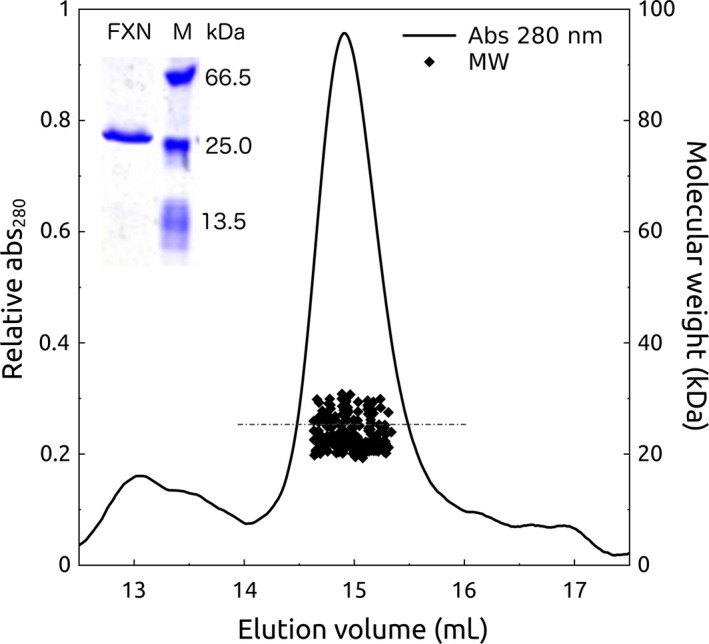
Hydrodynamic Behaviour of His6‐TAT‐FXN1‐210. Protein (14 μm) was run in a buffer of 20 mm Tris‐HCl, 600 mm NaCl, 1 mm EDTA, 1 mm DTT, 10% glycerol, at pH 7.5, in a MALS‐SEC‐FPLC system, followed by absorbance at 280 nm. The dashed line indicates the theoretical molecular weight corresponding to the full‐length His6‐TAT‐FXN1‐210 variant. The inset shows the result of a SDS/PAGE stained with coomassie blue analysis. Molecular weight markers (M) are: BSA (66 kDa), HPRT (25 kDa) and FXN90‐210 (13.5 kDa).

His6‐TAT‐FXN1‐210 was mainly monomeric, as inferred from MALS‐SEC‐FPLC experiments (Fig. 1), and the molecular weight matched with the theoretical one (dashed line), even though species of higher molecular weight were also observed in the SEC profile (at an elution volume of 13–13.5 mL). In addition, mass spectrometry (the experimental and theoretical masses were 25773.6 and 25768.2 Da, respectively) and SDS/PAGE analyses (inset, Fig. 1) indicated that the primary sequence corresponded to the one expected for His6‐TAT‐FXN1‐210. Therefore, no proteolytic cuts occurred during purification or during the experiment.

**Figure 2:**
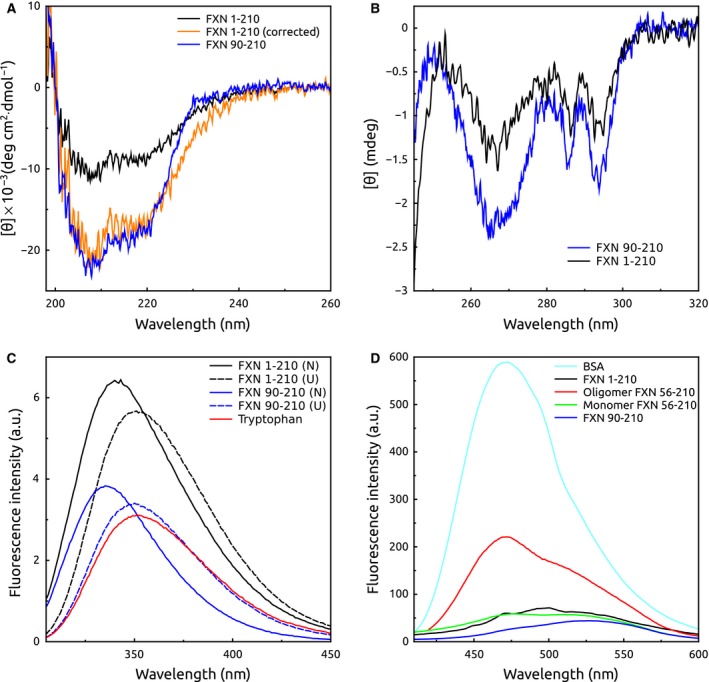
Spectroscopic Analysis of His6‐TAT‐FXN1‐210. (A) Far‐ and (B) Near‐UV CD spectra. Spectra corresponding to the precursor and FXN90‐210 are shown in black and blue, respectively. In addition, the spectra of the precursor calculated considering the length of the C‐terminal domain (90–210) is included (orange); (C) tryptophan fluorescence spectra were acquired for the precursor and FXN90‐210 (black and blue, respectively), excitation was performed at 295 nm, and slits correspond to 4 nm. U (unfolded conditions) and N (native conditions) indicate the presence or absence of 5.0 m GdmCl, respectively; (D) ANS binding was evaluated for the precursor (black) and FXN90‐210 (blue). As references, we evaluated binding of ANS to BSA (in cyan) and variant FXN56‐210 in two conformations, monomer and multimer, in green and red, respectively; the buffer was 20 mm Tris‐HCl, 300 mm NaCl, 1 mm EDTA, 1 mm DTT, 15% glycerol, at pH 7.5. All the experiments were carried out at 20 °C.

To determine the conformational properties of the recombinant precursor, we studied this protein by circular dichroism (CD) and fluorescence spectroscopy (Fig. 2). Far‐UV CD spectra were acquired (Fig. 2A). Results showed that His6‐TAT‐FXN1‐210 has a secondary structure content compatible with an α/β protein. When molar ellipticity was calculated assuming it to be full length, the CD signal was substantially lower than the one observed for the mature form of FXN; however, when ellipticity was calculated considering only the number of residues of the C‐terminal domain (90–210), spectra of both proteins superimposed very well, indicating that the N‐terminal region does not significantly contribute to the spectrum of the former and that the N‐terminal residues 1–89 are more likely unstructured. Analyses of near‐UV CD spectra of the precursor form (Fig. 2B) suggest that some changes in the local environment of aromatic residues may occur; yet, the addition of 1.0 m GdmCl did not produce significant changes in spectra features (Fig. S1), being the latter evidence of the integrity of the C‐terminal domain of the protein in this condition. More important, results suggest the absence of chiral environments for the aromatic side chains of the 1–89 stretch. In the same fashion, results suggest that some of the tryptophan residues that contribute to the fluorescence spectra of variant His6‐TAT‐FXN1‐210 are considerably exposed to solvent (Fig. 2C). This was inferred from the observed shift to the red in the value of λ_MAX_ (335 nm and 340 nm for FXN90‐210 and His6‐TAT‐FXN1‐210, respectively), and by comparison to tryptophan fluorescence spectra (352 nm) and spectra corresponding to GdmCl‐induced unfolded proteins (3 h of incubation in 3.7 m GdmCl, 351–352 nm). These results are compatible with a substantial solvation of tryptophan residues located in the N‐terminal region (Trp 2 and/or Trp 66) and a preserved C‐terminal domain.

On the other hand, the absence of ANS binding (Fig. 2D) indicates that N‐terminal residues 1–89 of His6‐TAT‐FXN1‐210 do not present hydrophobic pockets accessible to this dye as is commonly observed in molten globules or partially folded conformations.

### Conformational stability of FXN precursor and its aggregation tendency

**Figure 3:**
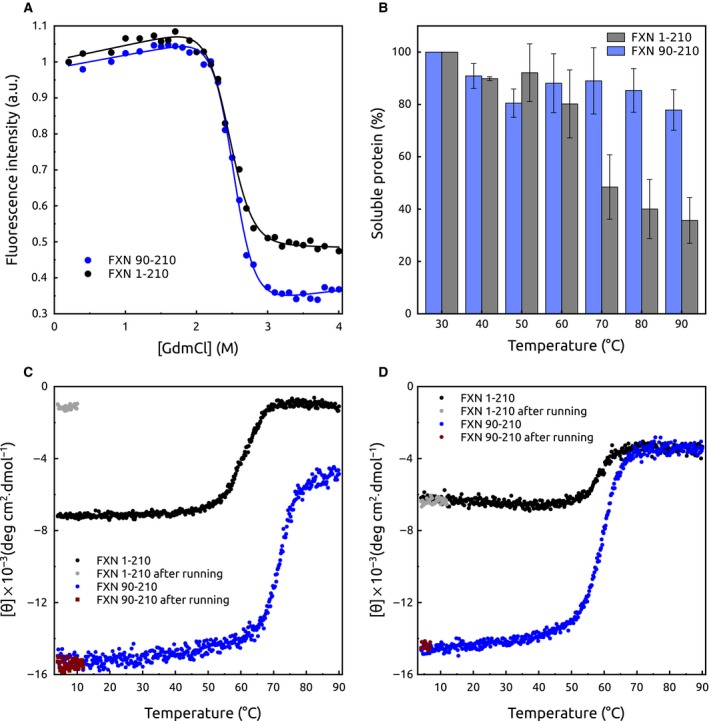
Unfolding and Stability of FXN Variants. (A) Protein unfolding was followed by tryptophan fluorescence intensity at 310 nm. Excitation was performed at 295 nm. After GdmCl addition, proteins were incubated for 3 h to guarantee equilibrium conditions. Native and unfolded fractions and m_NU_ and Δ*G*°_NU_ were calculated by fitting a two‐state model to the data. The buffer was 20 mm Tris‐HCl, 300 mm NaCl, 1 mm EDTA, 1 mm DTT, 15% glycerol, at pH 7.5. (B) Aggregation tendency upon controlled heating. The fraction of soluble protein was quantified by SDS/PAGE and coomassie blue staining. Temperature unfolding/aggregation followed by CD at 222 nm in the absence (C) or in the presence (D) of 1.0 m GdmCl. In temperature‐unfolding experiments, the buffer was 20 mm sodium phosphate, 100 mm NaCl, 0.1 mm EDTA, 0.1 mm DTT, 15% glycerol, at pH 7.5. CD signals obtained after temperature denaturation and a return to the initial temperature are shown in red and grey for FXN90‐210 and His6‐TAT‐FXN1‐210, respectively.

Thermodynamic stability of the His6‐TAT‐FXN1‐210 variant was researched by carrying out a GdmCl‐induced equilibrium unfolding experiment followed by tryptophan fluorescence (Fig. 3A). Although FXN90‐210 exhibited a folding intermediate state (it can be inferred from folding/unfolding kinetic experiments), a two‐state unfolding model can properly describe the equilibrium unfolding 26. Results showed that the precursor was as stable as FXN90‐210 and unfolding was cooperative (Table 1). The comparison between m_NU_ values obtained for the studied variants suggests that the transition observed corresponds to the unfolding of the C‐terminal domain of the precursor. This indicates that the N‐terminal domain is more likely unstructured at low denaturant concentrations. The fact that the GdmCl‐induced unfolding process was reversible (Fig. S2) suggests that low denaturant concentrations can solubilise the N‐terminal region, resulting in less strong intra‐ and intermolecule interactions. The aggregation tendency of the precursor form was also investigated. The protein was incubated at different temperatures and the aggregation profile was compared with that of FXN90‐210. Even though the former showed a higher tendency towards aggregation at higher temperatures than the latter, both proteins exhibited marked thermostability (Fig. 3B) in these buffer conditions.

**Table 1:** GdmCl‐induced unfolding of FXN variants.^a^

Variant	Δ*G*° _NU H2O_ (kcal·mol^−1^)	m _NU_ (kcal·mol^−1^ m ^−1^)	C_m_ (m)
FXN 90‐210	10.0 ± 0.5	3.9 ± 0.2	2.5 ± 0.3
His6‐TAT‐FXN1‐210	9.6 ± 0.7	3.8 ± 0.3	2.5 ± 0.5

a Isothermal unfolding experiments were carried out in 20 mm Tris‐HCl, 300 mm NaCl, 1 mm EDTA, 1 mm DTT, 15% glycerol, at pH 7.5. Measurements were done at 20 °C. A two‐state unfolding model was fitted to the data.

**Figure 4:**
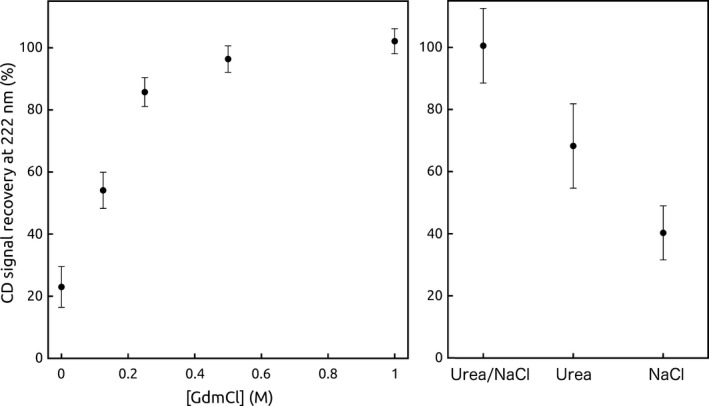
Reversibility of the Temperature‐Induced Unfolding of the Precursor as a Function of GdmCl Concentration. The recovery of the CD signal at 222 nm after returning to the initial temperature is quantified as the percentage of the starting CD signal, measured at 4 °C before starting the temperature ramp (left). The CD signal recovery under urea (2.0 m), NaCl (1.0 m) or the simultaneous addition of both at these concentrations is shown (right). The buffer was 20 mm sodium phosphate, 100 mm NaCl, 0.1 mm EDTA, 0.1 mm DTT, 15% glycerol, at pH 7.5. In the left panel, GdmCl was added to the indicated final concentrations, whereas in the right panel the same buffer was used, but instead of GdmCl, it was supplemented with urea 2.0 m and/or NaCl to 1.0 m final concentration.

It is worth mentioning that temperature‐induced unfolding/aggregation followed by CD spectroscopy showed an apparent *T*_m_ = 60 °C (Fig. 3C), a value that is significantly lower than the one observed for FXN90‐210 (*T*_m_ = 69.4 °C). In addition, unfolding of the recombinant precursor was not reversible (after heating, only ~ 20% of the CD signal at 222 nm was recovered when the sample returned to 4 °C, Fig. 4 and Fig. S3), thus hampering thermodynamic characterisation of this protein and indicating that the N‐terminal region affects foldability of the C‐terminal domain at high temperatures, regarding the mature form of FXN that unfolds in a reversible fashion. Remarkably, the apparent *T*_m_ value for His6‐TAT‐FXN1‐210 was more likely affected by an increased propensity for aggregation (Fig. 3C). In fact, the absence of the CD signal in the last part of the His6‐TAT‐FXN1‐210 denaturation profile (80–90 °C, Fig. 3C) indicated that most of the protein aggregated during the heating of the sample.

In agreement with the chemical unfolding experiment results, the addition of low concentrations of GdmCl (1.0 m) to the protein samples resulted in a completely reversible temperature‐induced unfolding process of His6‐TAT‐FXN1‐210 (Fig. 4 and Fig. S3) showing that the effects of the N‐terminal were minimised in these conditions. Moreover, the *T*_m_ value that characterises the unfolding transition of the precursor was practically the same as the one observed for FXN 90‐210 and the temperature‐unfolding profiles of both variants perfectly superimposed. On the other hand, lower GdmCl concentrations (in the range 0.25–0.50 m, Fig. 4 and Fig. S3) only resulted in a partial recovery of the CD signal at 222 nm after sample cooling, thus indicating that the N‐terminal domain established intra‐ or intermolecular interactions favouring FXN aggregation at high temperatures. It is worth noting that in these experiments the percentage of recovery depended on both the kinetic and thermodynamic aspects of aggregation and refolding, making it hard to interpret.

**Figure 5:**
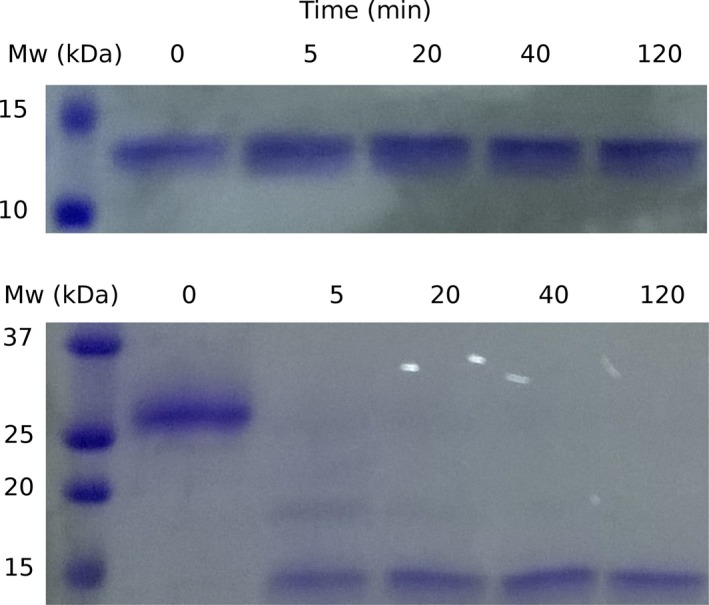
Sensitivity to Chymotrypsin of His6‐TAT‐FXN1‐210. Variant FXN90‐210 was included as a control. Incubation time (minutes) is indicated and the buffer was 20 mm Tris‐HCl, 300 mm NaCl, 1 mm EDTA, 1 mm DTT, 15% glycerol, at pH 7.5. For this experiment, a 1 : 100 protease to protein mass ratio was used.

To gain information on the structural dynamics of the variant, we studied the sensitivity to proteolysis of His6‐TAT‐FXN1‐210 (Fig. 5). After a short incubation with chymotrypsin (or trypsin, data not shown), the recombinant precursor was digested to a shorter form of ∼14.3 kDa, suggesting that the C‐terminal domain (residues 81–210) was resistant, whereas the N‐terminal was highly sensitive as in the case of FXN56‐210 24. On the other hand, as previously shown, the control variant FXN 90‐210 was completely resistant.

### The N‐terminal region of FXN is predicted as a disordered stretch

**Figure 6:**
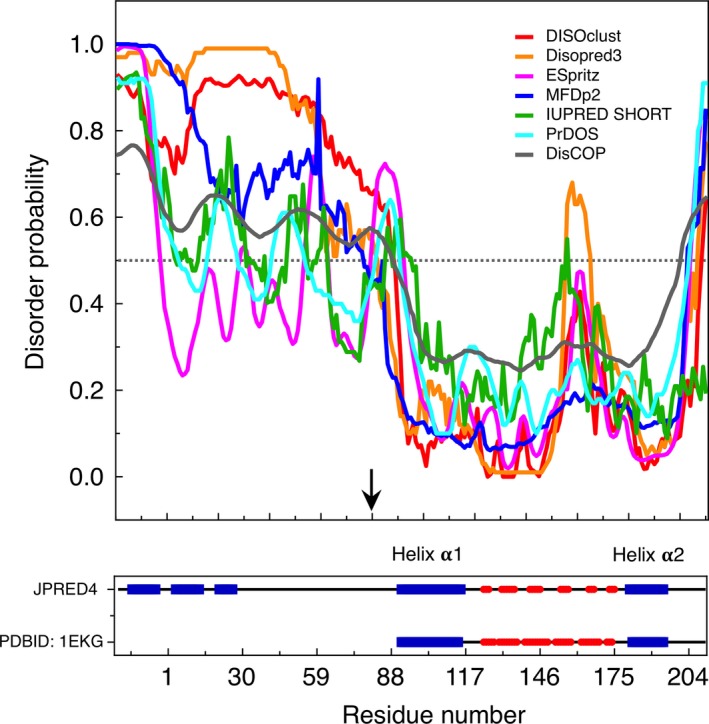
Predictions of Disorder in the N‐terminal Segment of His6‐TAT‐FXN1‐210 Precursor. Disorder predictions were carried out using a number of programmes: DiSOclust (red, 32), Disopred3 (orange, 30), Espritz (magenta, 31), MFDp2 (blue, 34), IUPRED (green, 35), PrDOS (cyan,^33^) and DisCOP (dark‐grey, 29). Disorder probability is plotted and the dotted line shows the 0.5 threshold. In the lower panel, the prediction of secondary structure (Jpred4 36) content is plotted side by side with the secondary structure content obtained from the X‐ray structure (PDB ID: 1EKG, 45). The black arrow indicates the starting residue of the mature form of FXN (FXN81‐210). Numbering corresponds to that of the precursor of FXN, for this reason His6‐TAT‐FXN1‐210 starts at residue ‐19.

Given that experimental evidence firmly suggested that the N‐terminal region is unstructured, we evaluated whether the complete N‐terminal polypeptide segment (residues 1–89) was predicted as disordered by bioinformatics tools 27, 28. As can be seen in Fig. 6, where the sequence of His6‐TAT‐FXN1‐210 was analysed, different predictors 29, 30, 31, 32, 33, 34, 35 coincided in the fact that the N‐terminal region may indeed be unstructured and similar results were obtained for the precursor FXN1‐210 (without the His6‐TAT N‐terminal stretch, Fig. S4). In addition, secondary structure predictions using JPred4 36 suggested that only very short helical segments may be immersed in the N‐terminal coil region of the human FXN. Our results are in good agreement with previous studies. On the one hand, the short N‐terminal segment of the mature form of FXN comprising residues 81 and 89 (recombinant variant FXN90‐210 lacks this segment) was completely unstructured, as judged by secondary chemical shifts 23; the recombinant protein FXN45‐210 (similar to the intermediate form FXN41‐210) was devoid of a periodic structure in the segments 41–81 22. In agreement with the latter, the N‐terminal region of the form FXN41‐210 (in this case, FXN was in the context of a multimeric protein assembly that included NFS) exhibited a nonperiodic structure, as judged by electron microscopy results 37. In this case, only some short segments showed a helical conformation (residues 52–57 and 68–69, PDB ID: 5KZ5). Remarkably, the unfolded nature of the N‐terminal segment was previously related to its functionality as a substrate during proteolytic processing 22, in the transit to the mitochondrial matrix.

### Activation of NFS1/ISD11/ISCU protein complex *in vitro*, interaction with cells and with the immune system

**Figure 7:**
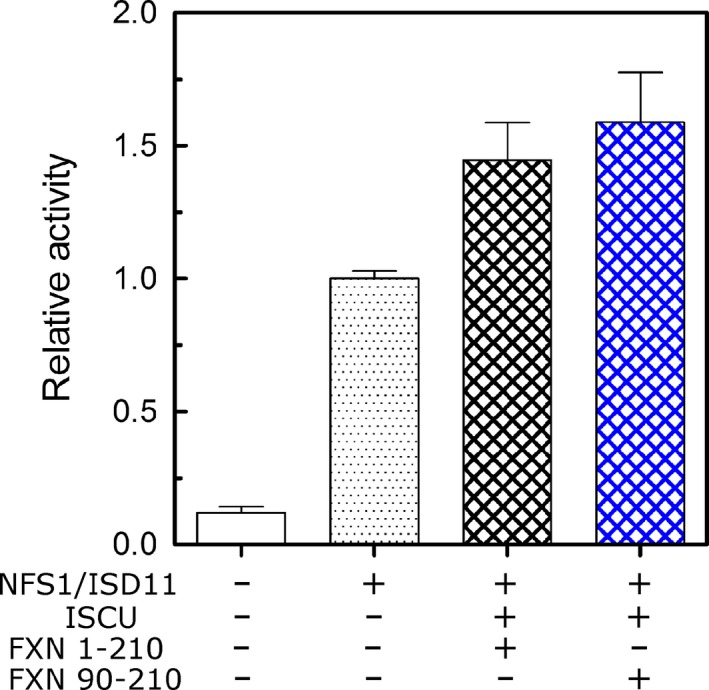
Activation of NFS1/ISD11/ISCU by His6‐TAT‐FXN1‐210. The reaction comprises the use of cysteine as a substrate, whereas in the presence of DTT the products are alanine and hydrogen sulfide. The detection of sulfide was carried out by the methylene blue method. The reaction was carried out in the presence of a combination of mouse NFS1/ISD11 (purified from *Escherichia coli*), human ISCU (purified from *E. coli*), and His6‐TAT‐FXN1‐210 or FXN90‐210 as a control. The specific combination is indicated using symbols + and – (presence and absence, respectively) and 10% of glycerol was added to the reaction buffer.

The activation of the NFS1/ISD11/ISCU protein complex by FXN is indeed one of the most relevant functions of FXN described to date. It can easily be followed by activation of desulfurase activity of the protein complex. Activation directly depends on the interaction of FXN with the protein complex and it is enhanced by the presence of Fe^+2^ 7, 38. Therefore, activation is an attractive probe for His6‐TAT‐FXN1‐210 structural dynamics. His6‐TAT‐FXN1‐210 produces desulfurase activation in a similar fashion as variant FXN90‐210. This fact suggests that, at least in the experimental conditions herein assayed, the N‐terminal extension was not detrimental from a functional view point (Fig. 7).

**Figure 8:**
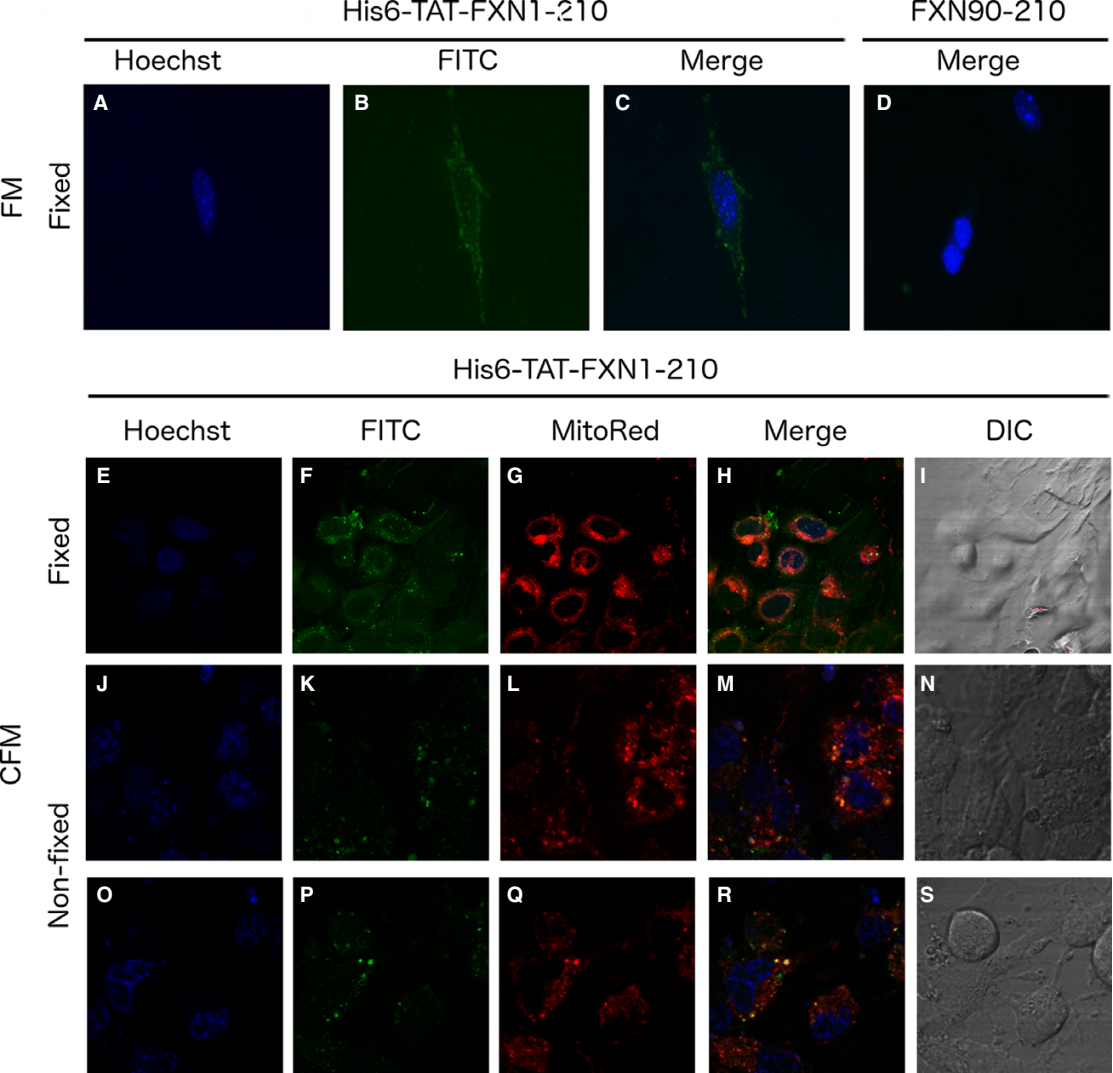
FICT‐Labelled His6‐TAT‐FXN1‐210 Interaction with Cells. The translocation of the FXN precursor across the cytoplasmic membrane using B104 cells was evaluated by fluorescence microscopy. 20 μg/mL of FICT‐labelled His6‐TAT‐FXN1‐210 (A, B and C) or FICT‐labelled FXN90‐210 (D) were added to the fresh culture medium and, after a 4 h‐incubation, cover slides were washed, fixed and stained with Hoescht. Subcellular localisation of the FICT‐Labelled His6‐TAT‐FXN1‐210 was investigated by CFM in fixed B104 cells in 4% paraformaldehyde, 10 min (E–I) and nonfixed B104 cells (J–S). Panels show Hoescht (E, J and O), FITC (F, K and P) or MitoRed (G, L and Q) fluorescence and merge of the three snapshots (H, M and R). Cell structure was inferred applying differential interference contrast (DIC, I, N and S). A 60× immersion oil objective was used. Panels J–N and O–S correspond to two different fields. A digital zoom (4× for fixed and not‐fixed cells) was applied.

We studied the translocation of the recombinant purified protein across the cytoplasmic membrane using mouse neuroblastoma cell line as a model. To achieve this, neuroblastoma B104 cells were cultured in the presence of FITC‐labelled His6‐TAT‐FXN1‐210 for 4–5 h at 37 °C, a period of time considered to be enough for protein internalisation. Nuclei were stained with Hoescht dye and outer membrane fluorescence was quenched using trypan blue. Fluorescence microscopy results showed a significant intracellular fluorescence pattern suggesting that the precursor protein not only exhibited a correctly folded C‐terminal domain, but also was able to translocate across cell membranes (Fig. 8A–C). On the other hand, FITC‐labelled FXN90‐210 was not able to interact in an efficient fashion with the cell culture (Fig. 8D).

To gain more information on the localisation of the FITC‐labelled recombinant variant inside the cell, we carried out confocal fluorescence microscopy (CFM) that allowed for an increase in resolution by blocking out‐of‐focus light in image construction. To infer whether the recombinant protein was guided to the mitochondria, cells were incubated with a MitoRed indicator reagent (Abcam, Cambridge, MA, USA), a cell‐membrane‐permeable rhodamine‐based fluorophore whose interaction with the organelle depends on the membrane potential and, once inside the mitochondria, is covalently trapped.

Results showed that FITC fluorescence was distributed in regions that superimposed with those regions revealed by MitoRed fluorescence, suggesting that the FITC‐labelled recombinant FXN variant reached mitochondria (Fig. 8E–I). Remarkably, cell fixing slightly altered the distribution of the recombinant protein by comparison with the unfixed cells, which showed clear‐cut results indicating an overlapping between FICT and MitoRed fluorescence (Fig. 8J–N).

**Figure 9:**
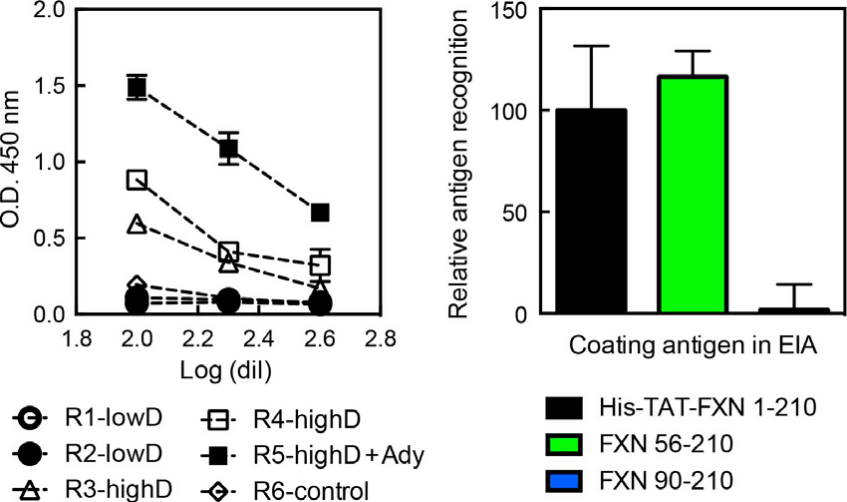
Immune Response against His6‐TAT‐FXN1‐210. (A) Mice were primed with His6‐TAT‐FXN1‐210 at a low dose (LowD, 0.05 nmol) or high dose (HighD, 0.25 nmol), and sera were titrated for the detection of specific antibodies anti His6‐TAT‐FXN1‐210. Two control groups were included: one without protein and another one with an adjuvant. (B) Specific antibodies to His6‐TAT‐FXN1‐210, FXN90‐210 and FXN56‐210 were tested by ELISA.

As His6‐TAT‐FXN1‐210 has been proposed as an exogenous construct to be used as a substitute for the physiological FXN (which is absent or decreased in FRDA patients), another important issue was whether His6‐TAT‐FXN1‐210 was immunogenic, given that replacement therapies are applied for life, and considering that the TAT sequence is a viral‐derived peptide. To test this, we evaluated the specific antibody response after a single injection of the precursor in two doses. Serum was extracted and titrated against FXN90‐210, FXN56‐210 or His6‐TAT‐FXN1‐210 (Fig. 9) by ELISA. Only a mild antibody‐mediated immune response was detected, and this response was mainly directed against the N‐terminal stretch, as suggested by the absence of reactivity detection when FXN90‐210 was used in the test. On the other hand, similar responses were observed for FXN56‐210 or His6‐TAT‐FXN1‐210. At this moment, we cannot rule out a specific, but weak, response against the TAT peptide or His tag, and more experiments should be conducted. However, results support the notion that the C‐terminal fragment of the construction (residues 90–210) is not immunogenic. Regarding the immune response observed against the N‐terminal of FXN56‐210, it is important to consider that this region of the protein concentrates most of the differences existing between mouse and human FXN sequences and this might be one of the reasons for its immunogenicity. It is worth to mention that one of the main features of the N‐terminal stretch 1–89 is the low conservation of this segment by comparison to the highly conserved FXN domain 90–210 (Figs. S5 and S6).

### Ubiquitination of His6‐TAT‐FXN1‐210

**Figure 10:**
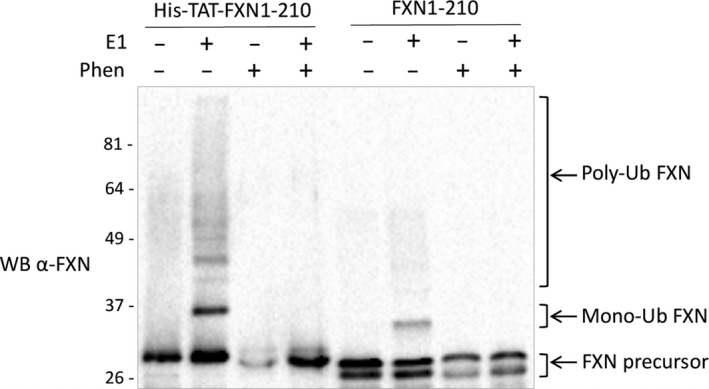
Ubiquitination of His6‐TAT‐FXN1‐210. An *in vitro* ubiquitination assay was carried out with purified recombinant human E1, UbcH5b as the E2 ubiquitin‐conjugating enzyme, and GST‐ RNF126 as the E3 ubiquitin ligase, together with Ub, ATP, and recombinant human His6‐TAT‐FXN1‐210 or FXN1‐210 as a substrate. Where indicated, 10 mm of 1,10‐phenanthroline, the Zn chelating agent, was added to the reaction. The reaction mixture was incubated for 60 min at 30 °C. Reaction was stopped by the addition of a Laemli sample buffer. Proteins were separated on SDS/PAGE and analysed by western blotting with an anti‐frataxin antibody. The FXN precursor, monoubiquitinated FXN, and polyubiquitinated FXN are indicated by arrows.

A significant portion of the FXN precursor undergoes ubiquitination, mediated by the recently identified E3 ligase RNF126, before reaching the mitochondria. This event leads to proteasomal degradation of the precursor, negatively affecting the amount of mature FXN generated 39. To determine whether His6‐TAT‐FXN1‐210 was susceptible to ubiquitination and degradation as the endogenous precursor, we tested the ability of the E3 ligase RNF126 to ubiquitinate this FXN variant *in vitro*, using an established FXN ubiquitination assay 39. As shown in Fig. 10, when His6‐TAT‐FXN1‐210 was incubated with the E1, the E2 and RNF126 as the E3 ligase, the anti‐frataxin antibody detected slower migrating bands by western blot, indicating that His6‐TAT‐FXN1‐210 can be ubiqutinated as observed for FXN1‐210. Moreover, ubiquitination of His6‐TAT‐FXN1‐210 was prevented by the addition of the Zn^2+^ chelating agent 1,10‐phenanthroline, as described for FXN1‐210 39, suggesting that His6‐TAT‐FXN1‐210 ubiquitination is dependent on RNF126 catalytic activity. These experimental data suggest that structural features, which are necessary for FXN recognition by RNF126, are preserved in the His6‐TAT‐FXN1‐210 variant and, more important, the His6‐TAT extension does not interfere with the ubiquitination machinery.

## Discussion

His6‐TAT‐FXN1‐210 was expressed in *E. coli* and purified under native conditions. The protein principally behaved as a monomer with its C‐terminal domain compact and structured. However, its N‐terminal domain showed an extremely low conservation score compared to the C‐terminal domain (Fig. S4) and it was disordered and extremely sensitive to proteolysis. Even though His6‐TAT‐FXN1‐210 had an increased propensity to aggregate by comparison with the mature form, conditions were set in which the protein was stable at 4 °C and the precursor remained soluble after freeze and thaw, and auto‐proteolysis was minimised. In addition, the incubation of the FXN precursor with low GdmCl concentration hampered N‐terminal‐mediated aggregation and promoted *in vitro* the reversible unfolding of the C‐terminal domain. Although we inferred that the thermodynamic stability of the C‐terminal domain of the precursor variant His6‐TAT‐FXN1‐210 was similar to that of the mature form, at this time we cannot rule out that the N‐terminal stretch has a role in folding kinetics.

One of the most noticeable features of the FXN structure is the large anionic surface formed by the Glu and Asp residues located in the acidic ridge motif (helix α1, loop‐1 and strand β1). Given that the N‐terminal region formed by residues 1–89 exhibited a substantial number of positively charged residues, one simple explanation for the aggregation propensity was that electrostatic intra‐ and intermolecular interactions between both regions were the driving force. The presence of disorder segments may make this interaction process more efficient by adding a certain conformational freedom, allowing for the exploration of a larger conformational space. Furthermore, as the cell‐penetrating peptide TAT stretch was highly positive, we suggest that electrostatic interactions may become stronger in His6‐TAT‐FXN1‐210 than in the wild‐type FXN precursor. In this context, it was reasonable to conclude that the addition of 1.0 m GdmCl completely prevented these interactions, making the subsequent temperature‐unfolding process reversible, whereas 2.0 m urea or 1.0 m NaCl were less efficient and aggregation occurred. Remarkably, the simultaneous addition of both urea and NaCl at these concentrations allowed for 100% of reversibility (Fig. 4); on the one hand, this was indicative of the key role of electrostatics in the establishment of interactions that resulted in aggregation and, on the other hand, it suggested the contribution of apolar contacts to protein–protein interaction. In this context, it will very important to carry out aggregation and refolding measurements varying pH to test the involvement of ionisable groups in the aggregation process of His6‐TAT‐FXN1‐210. Evidence of intermolecular interaction in human FXN was provided by the FXN56‐210 variant, which exhibited a high aggregation propensity. In fact, it formed large soluble and stable oligomers at room temperature 24 and the unstructured N‐terminal region mediated this interaction. When the N‐terminal was removed by proteolysis the oligomer disassembled.

Frataxin oligomers have been proposed as playing a role *in vivo*, and a low resolution (14.3 Å) cryoelectron microscopy structure of a high‐molecular weight multimolecular complex involving human NFS1/ISD11, ISCU and FXN (PDB ID: 5KZ5, the proteins responsible for the iron‐sulfur cluster assembly) was recently published 37. Trimers formed by FXN42‐210 subunits interacted through their N‐terminal stretches. Short α‐helical regions (residues 52–58) and a high content of nonperiodic structure established several intermolecular contacts. It is worth noting that interaction was also mediated by contacts established by numerous residues located in the C‐terminal domain, thus forming a large contact surface, which suggests a cooperative assembly. Interesting information concerning desulfurase activity of the NFS1/ISD11/ISCU/FXN42‐210 protein complex was obtained showing that FXN42‐210 was active 37.

The fact that His6‐TAT‐FXN1‐210 was also functional as the activator of the desulfurase complex NFS1/ISD11/ISCU suggests that conformational features were preserved in this variant and that the N‐terminal part of the protein did not produce steric clashes (in the context of the multiprotein complex) that might have resulted in a significant loss of FXN functionality.

It is also worth noting that His6‐TAT‐FXN1‐210 was recognized and ubiquitinated by the recently identified frataxin E3 ligase, RNF126. These experimental data indicated that structural requirements for interaction between frataxin and E3 ligase were maintained in the His6‐TAT‐FXN1‐210 variant. However, since it is known that ubiquitination leads to proteasomal degradation of FXN precursor and, in turn, results in a reduced amount of the mature FXN generated, a FXN variant that loses the ability to interact with RNF126 is expected to yield a more mature and functional FXN. With the aim of obtaining an efficient tool for protein delivery therapeutics, it may be possible to envision the generation of such an ‘ubiquitin‐resistant’ FXN variant.

Important studies of other groups have shown that TAT constructs have a key effect on the mitochondrial metabolism, suggesting that FXN locates inside the organelle and can restore mitochondrial function. In this way, Vyas *et al*. used TAT‐FXN fusion protein to deliver human FXN to mitochondria both in cultured patient cells and in a severe mouse model of FRDA, increasing lifespan and cardiac function in the latter 13. In addition, Britti *et al*. studied the effect of the TAT‐FXN variants on the progression of neurodegeneration markers on frataxin‐depleted neurons. Also, they investigated the ability of TAT‐FXN to reach muscle mitochondria by restoring the enzymatic activity levels of the succinate dehydrogenase 40. Furthermore, Kim *et al*. showed the capability of TAT‐FXN to cross the blood‐brain barrier protecting neurons against oxidative stress in mouse models 14. Moreover, Marcus *et al*. studied several heterologous mitochondrial targeting sequences (MTS) for delivering FXN. They showed that the level of functional FXN in the mitochondria is higher than the one observed when native MTS sequences are used, and the result was supported by the rescue of FRDA cells from oxidative stress and the observation of the increase in aconitase enzymatic activity and ATP levels, compatible with a restoration of mitochondrial function 12.

In this context, we hope that our results, which show that the purified His6‐TAT‐FXN1‐210 variant was thermodynamically stable and exhibited a well‐folded C‐terminal domain that was functional as a desulurase NFS1/ISD11 activator, will help to delineate the strategy for FXN precursor production and its conformational evaluation, an important aspect in protein replacement therapy. In this regard, the propensity for aggregation that is observed for the recombinant variant at high temperatures should be taken into account in future works.

## Materials and methods

### Expression and purification of the FXN variants

Variants FXN90‐210 and His6‐TAT‐FXN1‐210 were expressed in *Escherichia coli* codon plus ROSETTA2pLysDE3 BL21(DE3) cells, and purified from the soluble fractions under native condition. Variant FXN90‐210 was prepared as previously 26, 41. On the other hand, variant His6‐TAT‐FXN1‐210 was purified to ≥ 95% (checked by SDS/PAGE). Briefly, His6‐TAT‐FXN1‐210 expression was induced with 1% lactose for 3 h, at 250 r.p.m. and 37 °C. Cells were harvested by centrifugation (at 5800 ***g*** for 15 min at 4 °C) and stored at −20 °C until use. Typically, 3 g of cells were disrupted by a French Press in 20 mm Tris‐HCl buffer, 300 mm NaCl, 0.02% (v/v) Tween 20 and 20 mm imidazole, at pH 7.5. Soluble and insoluble fractions were analysed by SDS/PAGE. His6‐TAT‐FXN1‐210 was purified from the supernatant by Ni^2+^‐NTA‐agarose chromatography using steps of 20, 50, 100, 200 and 500 mm imidazole for the elution. Most of the recombinant protein typically elutes with 500 mm imidazole. After that, His6‐TAT‐FXN1‐210 was extensively dialysed against 20 mm Tris‐HCl, 300 mm NaCl, 1 mm EDTA, 1 mm DTT, at pH 7.5 to remove imidazole. Before freezing, 15% glycerol was added to the protein. To confirm the expected masses of the proteins, ESI‐MS (Thermo Finnigan) was performed. In addition, when DTT was removed using a PD10 column, two thiols per protein molecule were detected by reaction with 5,5′‐dithiobis‐(2‐nitrobenzoic acid), indicating the absence of intra‐ or intermolecular disulfide bonds interactions.

### Size‐exclusion chromatography and light‐scattering measurements

Mass determination was performed by multiple‐angle laser light‐scattering (MALLS) using miniDawn (Wyatt Technology, Santa Barbara, CA, USA) coupled to a size‐exclusion Superose‐12 column (GE Healthcare, Chicago, IL, USA). Protein concentration was 10 μm and the elution buffer was 20 mm Tris‐HCl, 600 mm NaCl, 1 mm EDTA, 15% glycerol, at pH 7.5. A concentration of 600 mm NaCl prevented the interaction of the protein with the chromatographic media. The experiments were carried out at room temperature (~ 25 °C) at a 0.2–04 mL·min^−1^ flow rate. Data analysis was performed using the Astra 6.0 software (Wyatt Technology).

### Spectroscopic characterisation of the FXN variants

Circular dichroism (CD) measurements were carried out with a Jasco J‐810 spectropolarimeter. Near‐UV and far‐UV CD spectra were collected using cells of 1.0 and 0.1 cm path length respectively. Data were acquired at a scan speed of 20 nm·min^−1^ and at least 10 scans were averaged. For near‐UV CD spectroscopy, protein concentration was 10 μm.

Steady‐state intrinsic fluorescence measurements were performed in a Jasco FP‐6500 spectrofluorometer operating in the ratio mode. A 1.0‐cm path‐length cell was used. Intrinsic fluorescence of proteins was measured using a protein concentration of 2 μm; excitation wavelength was 295 nm and emission data were collected in the range of 305–450 nm. The spectral slit‐widths were set to 3 nm for both monochromators. Experiments were performed using a thermostated cell holder connected to a circulating water bath set at 20 °C. Buffer was 20 mm Tris‐HCl, 300 mm NaCl, 1 mm EDTA,1 mm DTT, 15% glycerol, at pH 7.5.

For 8‐anilino‐1‐naphthalene sulfonic acid (ANS)‐binding experiments, proteins (5 μm) in buffer, 20 mm Tris‐HCl, 300 mm NaCl, 1 mm EDTA,1 mm DTT, 15% glycerol, at pH 7.5 were incubated at room temperature (10 min), with 50 μm of ANS. Control samples consisting of ANS (50 μm) in buffer solution were prepared. As a positive control, 5 μm BSA was also incubated with the dye in the same conditions. The excitation wavelength was 350 nm and the emission spectra were collected between 400 and 600 nm. The bandwidth used was 3.0 nm for both excitation and emission. To estimate the concentration of the dye, the extinction coefficient ε_M_ = 4950 m ^−1^·cm^−1^ at 350 nm was used.

### Equilibrium unfolding experiments

Unfolding experiments (isothermal) were carried out incubating protein variants for 3 h at room temperature with 0–4.0 m GdmCl in a buffer 20 mm Tris‐HCl, 300 mm NaCl, 1 mm EDTA, 1 mm DTT, 15% glycerol, at pH 7.5. Measurements were done at 20 °C. The unfolding process was followed by tryptophan fluorescence. To determine thermodynamic parameters, the free energy of unfolding and its dependence with denaturant concentration (Δ*G*°_NU_ and m_NU_, respectively), we fitted a two‐state unfolding model to the data, where only native (N) and unfolded (U) conformations existed in equilibrium (U↔N). Data were processed according to Santoro and Bolen 42. All GdmCl concentrations were corroborated using a refractometer. Unfolding transitions as a function of temperature were monitored by the circular dichroism signal at 222 nm using a bandwidth of 2 nm. Experiments were carried out in 20 mm sodium phosphate, 100 mm NaCl at pH 7.5. Protein concentration was 1.5 μm. Temperature was varied from 4 to 90 °C, at a constant rate of 1 °C·min^−1^, sampling at 1 °C intervals and a 1.0 cm cell path length was used.

### Determination of cysteine desulfurase activity

The enzymatic desulfurilation of cysteine to alanine and sulfide by NFS1/ISD11 complex was determined by the methylene blue method 43. Reactions were carried out in a volume of 0.4 mL, containing 1 μm of NFS1/ISD11, 3 μm FXN and 3 μm ISCU. Samples were supplemented with 10 μm PLP, 2 mm DTT and 1 μm Fe^+2^ (final concentrations) and the reaction buffer was 50 mm Tris‐HCl, 200 mm NaCl, 10% glycerol, at pH 8.0. The buffer was degassed using argon. The reaction was started by the addition of 1 mm cysteine and samples were incubated at room temperature for 30 min. Sulfide production was stopped by the addition of 50 μL of 20 mm *N*,*N*‐dimethyl *p*‐phenylenediamine in 7.2 N HCl and 50 μL of 30 mm FeCl_3_ were prepared in 1.2 N HCl. Under these conditions, methylene blue production took 20 min. After that, the samples were centrifuged for 5 min at 12 000 ***g*** and supernatant was separated. Absorbance at 670 nm was measured.

### FITC‐Labelling of His6‐TAT‐FXN1‐210, cell culture and protein internalisation

For FICT‐labelling, His6‐TAT‐FXN1‐210 or FXN90‐210 proteins were incubated in the presence of FITC for 2 h at 24 °C in buffer 20 mm HEPES 300 mm NaCl, 1 mm EDTA, at pH 8.0. Protein and FITC concentrations were 0.38 and 0.038 μg·μL^−1^, respectively. Reactions were stopped by the addition of 100 mm Tris‐HCl (final concentration), at pH 7.6. After reaction, the free FITC was removed from protein solution by an extensive overnight dialysis at 4 °C against a buffer of 20 mm Tris‐HCl 300 mm NaCl, 1 mm EDTA, at pH 7.6. Proteins were centrifuged for 20 min at maximum speed using a refrigerated (4 °C) Eppendorf centrifuge and maintained at 4 °C until use.

B104 cells (ATCC CRL 1887) were cultured in RPMI 1640, in the presence of 10% FBS, penicillin–streptamycin (10 U·mL^−1^ each), glutamine (2 mm), up to a 50% of confluence, in sterile round cover slides in a 24‐well culture microplate. FITC‐labelled His6‐TAT‐FXN1‐210 was added to fresh culture medium at a concentration 20 μg·mL^−1^, and cells were incubated at 37 °C and controlled pCO_2_ for 4 h. The cover slides were then washed, fixed with ice‐cold methanol during 5 min, washed again and stained with Hoescht (10 μg·mL^−1^ during 2 min). Cover slides were extensively washed, dried and mounted using mowiol. Finally, cells were observed by fluorescence microscope (Olympus BX50). Subcellular localisation was investigated by confocal fluorescence microscopy (CFM). Mitochondria were identified using a MitoRed indicator reagent (Abcam). Given that it was previously suggested that cell‐fixing processes can alter the distribution of proteins that include a TAT sequence (in particular, it was observed that part of TAT‐including proteins seemed to appear in the nuclei, 44), two different strategies were followed:

- Cells were incubated with His6‐TAT‐FXN1‐210. After gentle washing, cells were incubated first with MitoRed (150 μL, 0.1×) for 30 min. After new washings, cells were incubated with Hoescht dye (20 μL, 10 μg·mL^−1^) for 5 min. Finally, cells were carefully washed and subsequently fixed (4% paraformaldehyde, 10 min) and mounted using mowiol;
- On the other hand, cells were grown in a 96‐multiwell plate suitable for fluorescence microscopy, incubated in the same way, first with the FITC‐labelled recombinant protein and then with fluorescent dyes; but in this latter case, cells were not fixed and MitoRed concentration was lower (0.015×).

In all cases, cells were observed using a confocal microscope FV1000 instrument (Olympus) and a 60× immersion oil objective. In addition, a digital zoom (4 and 3×, for fixed and not‐fixed cells, respectively) was applied.

### Ethics statement

The use of laboratory animals in this study was in accordance with the relevant guidelines and institutional policies, and the protocol was approved by the Animal Care and Use Committee at the School of Pharmacy and Biochemistry of the University of Buenos Aires (Argentina). All efforts were made to minimise the animal distress.

### Immune response measurements against TAT‐His6‐FXN1‐210

C57 male mice ranging from 8 to 12 weeks were used. The animals were kept in an environment with a constant temperature of 22 °C and 12‐h light–dark cycle (08:00–20:00), with food and water *ad libitum*. Mice were primed with His6‐TAT‐FXN1‐210 at a low dose (0.05 nmol) or high dose (0.25 nmol), according to previous experiences with other proteins. Proteins were intraperitoneal injected in sterile PBS at a final volume of 200 μL. Two control groups were included: one without protein and another one with adjuvant (0.04 g·mL^−1^ of aluminium hydroxide and 0.04 g·mL^−1^ of magnesium hydroxide). Animals were bled on days 15, 30 and 45, and specific antibodies to His6‐TAT‐FXN1‐210, FXN90‐210 and FXN56‐210 were tested by ELISA. Briefly, microplates were coated with the corresponding antigen, blocked with nonfat milk (0.03 g·mL^−1^ prepared in PBS) and then washed to remove nonbound proteins. Sera were titrated in twofold dilutions starting at 1/100, and specific antibodies were detected using anti‐mouse HRP labelled antibodies (Sigma‐Aldrich, St. Louis, MO, USA) at a final dilution of 1/5000. Peroxidase activity was detected using TMB substrate solution and the reaction was stopped with 4 N H_2_SO_4_. Absorbance was measured at 450 nm using a Tecan reader.

### *In vitro* ubiquitination assay

The *in vitro* ubiquitination assay was performed in a reaction mixture containing 100 nm of bacterially purified human recombinant His‐tagged E1, 1.4 mm of a human recombinant untagged E2 UbcH5b and 200 nm of human purified recombinant glutathione *S*‐transferase (GST)‐RNF126 (E3) in a ubiquitination buffer as described by Benini and coworkers 39. The human recombinant FXN1‐210 protein expressed in *E. coli* was purchased from GenScript. Recombinant ubiquitin was purchased from Enzo Life Sciences. Human recombinant GST‐RNF126 protein, purified using an *in vitro* wheat germ expression system, was purchased from Abnova. 1,10‐Phenanthroline monohydrochloride monohydrate (1,10‐phenanthroline) was purchased from Sigma‐Aldrich and, where indicated, was preincubated with RNF126 for 5 min before starting the assay. After incubation for 60 min at 30 °C, the reactions were terminated by adding 4× Laemmli sample buffer, loaded on a 10% Mini‐Protean TGX Stain‐Free Gel (Bio‐Rad) and followed by western blotting with a monoclonal anti‐frataxin antibody (clone 18A5DB1, Abcam).

## Author contributions

IHC, AF, MGH, MEN, LM and MB planned and performed the experiments, and analysed the data. AR, RT, PC and JS planned the experiments, analysed the data and wrote the paper.

## Supporting information


**Fig. S1.** Effect of 1.0 m GdmCl Concentration on the Tertiary Structure of the C‐Terminal Domain of His6‐TAT‐FXN1‐210.
**Fig. S2.** Reversibility of the GdmCl‐Induced Unfolding of the Precursor Followed by Tryptophan Fluorescence.
**Fig. S3.** Reversibility of Temperature‐Induced Unfolding of the Precursor as a Function of GdmCl Concentration.
**Fig. S4.** Predictions of Disorder in the N‐terminal Segment of FXN1‐210 Precursor.
**Fig. S5.** The N‐terminal of FXN1‐210 is not conserved along the evolution.
**Fig. S6.** The N‐terminal of FXN1‐210 is not conserved.
