# Supplementary material for: Biophysical characterisation of the recombinant human frataxin precursor
Source: FEBS Open Bio. 2018 Jan 25;8(3):390–405. doi: 10.1002/2211-5463.12376 (PMC5832983; doi:10.1002/2211-5463.12376)
Supplement: Supplementary file 1 — Fig. S1. Effect of 1.0 m GdmCl Concentration on the Tertiary Structure of the C‐Terminal Domain of His6‐TAT‐FXN1‐210. Fig. S2. Reversibility of the GdmCl‐Induced Unfolding of the Precursor Followed by Tryptophan Fluorescence. Fig. S3. Reversibility of Temperature‐Induced Unfolding of the Precursor as a Function of GdmCl Concentration. Fig. S4. Predictions of Disorder in the N‐terminal Segment of FXN1‐210 Precursor. Fig. S5. The N‐terminal of FXN1‐210 is not conserved along the evolution. Fig. S6. The N‐terminal of FXN1‐210 is not conserved. [file FEB4-8-390-s001.pdf]

Supplementary Material

Biophysical Characterisation of the Recombinant Human Frataxin Precursor

Ignacio Hugo Castro,[1] Alejandro Ferrari,[1] María Georgina Herrera,[1] Martín Ezequiel Noguera,[1] Lorenzo Maso,[2] Monica Benini,[3,4] Alessandra Rufini,[3,4] Roberto Testi,[3,4] Paola Costantini[2] and Javier Santos*[1]

[1]Institute of Biological Chemistry and Physical Chemistry (IQUIFIB) Dr. Alejandro Paladini, University of Buenos Aires, Junín 956, (C1113AAD), Buenos Aires, Argentina

[2]Department of Biology University of Padova, Viale G. Colombo 3, 35131, Padova, Italy

[3]Laboratory of Signal Transduction, Department of Biomedicine and Prevention, University of Rome ''Tor Vergata,'' Via Montpellier 1, 00133 Rome, Italy

[4]Fratagene Therapeutics srl, Viale dei Campioni 8, 00144, Rome, Italy

***Corresponding Authors:**

Javier Santos. Institute of Biological Chemistry and Physical Chemistry, University of Buenos Aires, Junín 956, 1113AAD, Buenos Aires, Argentina. Telephone: +54 114 964 8289 ext. 108, Fax: +54 114 962 5457. E-mail: javiersantosw@gmail.com

Running Title: The Frataxin Precursor

Keywords: Friedreich's Ataxia, precursor, stability, conformation, aggregation, unfolding

Abbreviations: CD, circular dichroism; CTR, C-terminal region; FITC, fluorescein isothiocyanate; FXN, frataxin; FRDA, Friedreich's Ataxia; GdmCl, guanidinium chloride; His6-TAT-FXN1-210, the precursor of FXN including an N-terminal His tag and a cell-penetrating peptide; TAT, trans-activator of transcription from HIV-1).

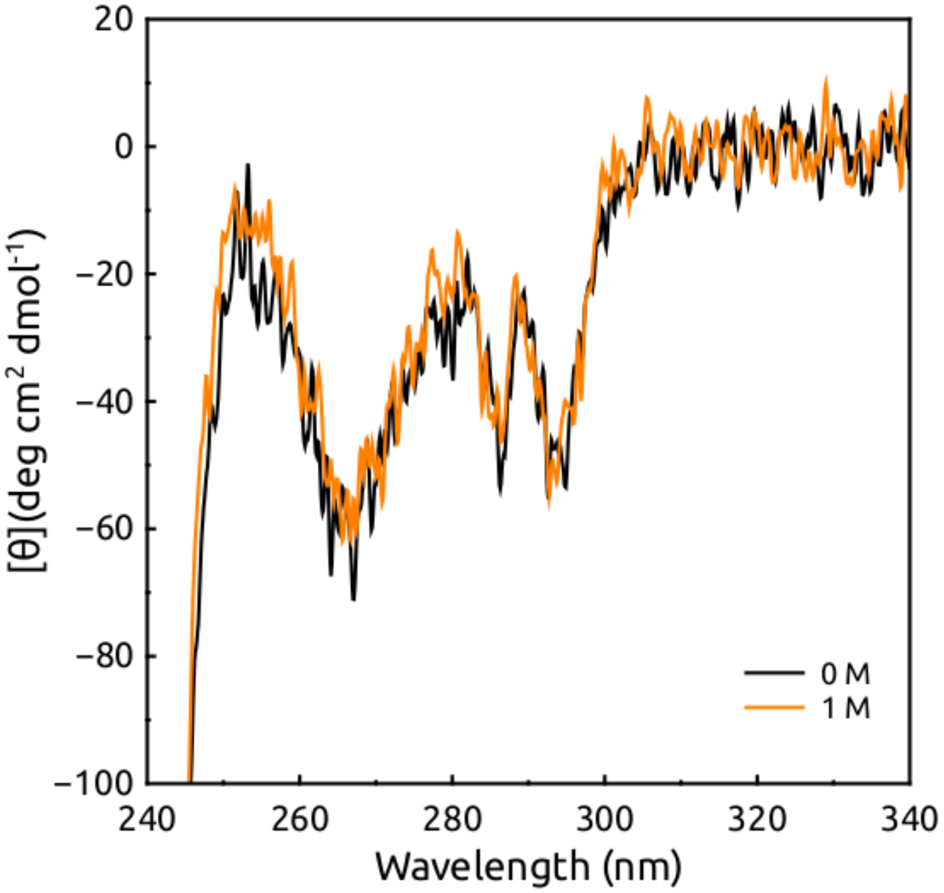

Figure S1. Effect of 1.0M GdmCl Concentration on the Tertiary Structure of the C-Terminal Domain of His6-TAT-FXN1-210. CD spectra in the near-UV was acquired at 25° C in the absence (black) and in the presence (orange) of 1.0M GdmCl. The buffer was 20 mM sodium phosphate, 100 mM NaCl, 0.1 mM EDTA, 0.1 mM DTT, 15 % glycerol, at pH 7.5.

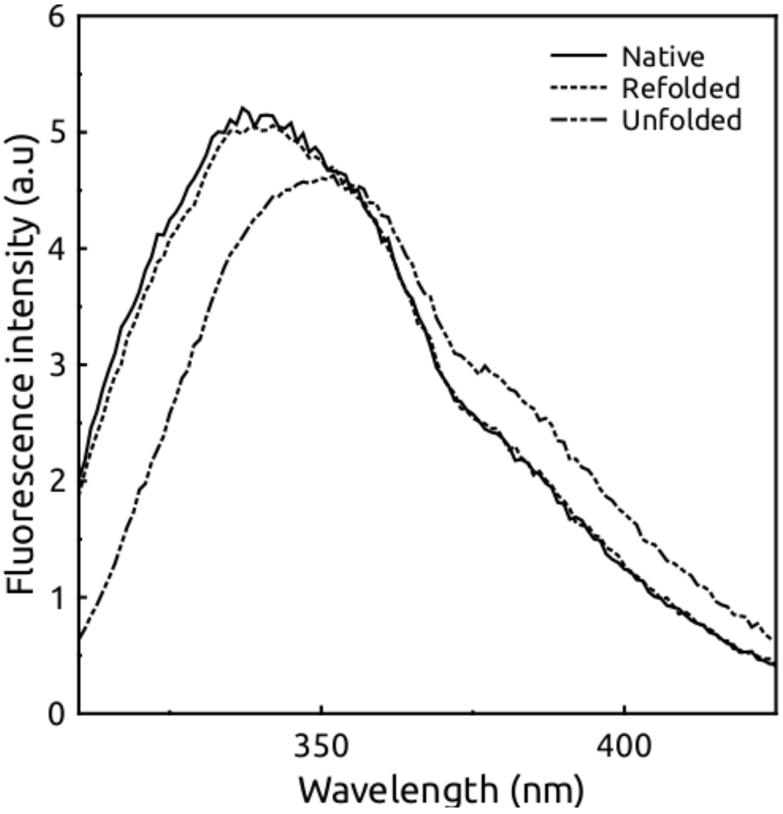

Figure S2. Reversibility of the GdmCl-Induced Unfolding of the Precursor Followed by Tryptophan Fluorescence. Spectra corresponding to His6-TAT-FXN1-210 in the presence (- - —) or in the absence (——) of GdmCl and spectra corresponding to a protein sample refolded by an over-night dialysis (------) are shown. The buffer was 20 mM sodium phosphate, 100 mM NaCl, 0.1 mM EDTA, 0.1 mM DTT, 15 % glycerol, at pH 7.5. Excitation was performed at 295 nm, and slits of 4 nm were used for both excitation and emission.

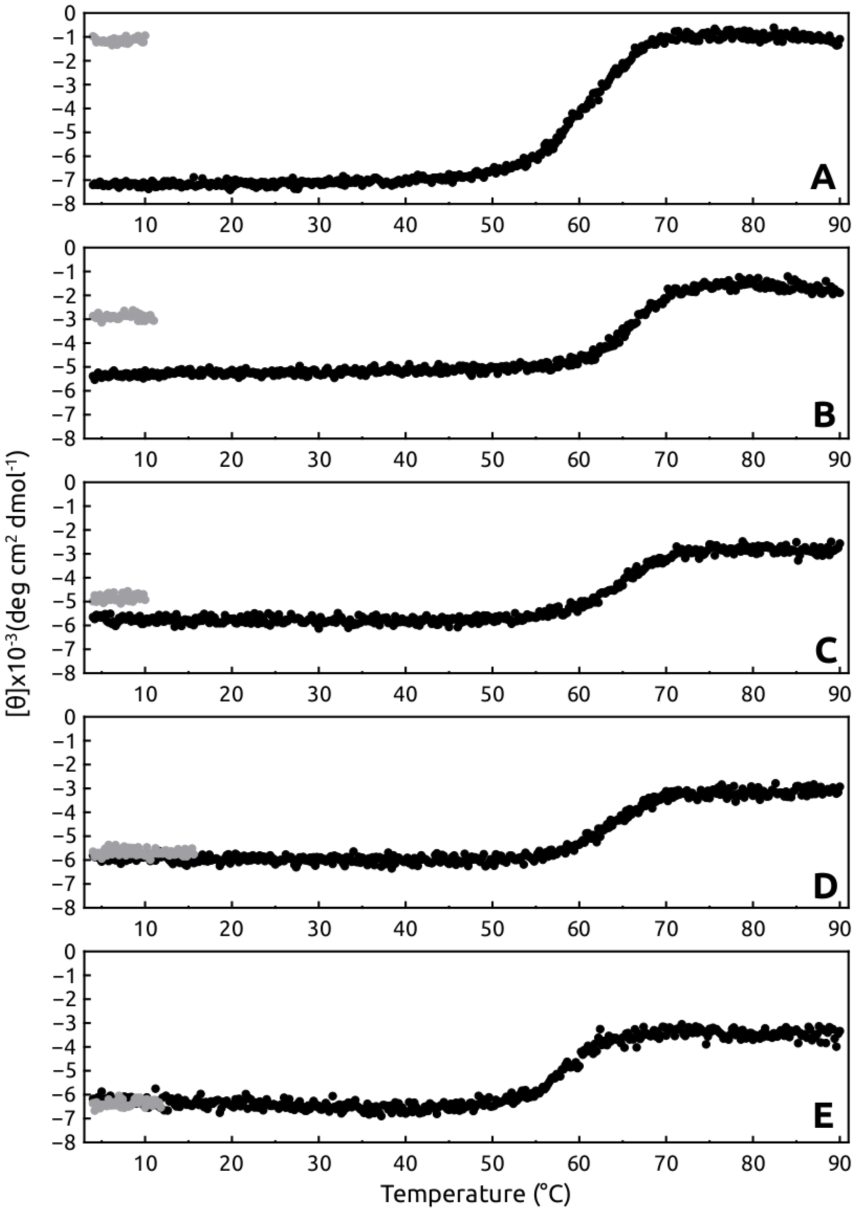

Figure S3. Reversibility of Temperature-Induced Unfolding of the Precursor as a Function of GdmCl Concentration. Denaturation profile (black) followed by a CD signal at 222 nm and the recovered CD signal (gray) after returning to the initial temperature (~5 min) and starting the ramp again are shown. The buffer was 20 mM sodium phosphate, 100 mM NaCl, 0.1 mM EDTA, 0.1 mM DTT, 15% glycerol, at pH 7.5. Concentrations of 0, 0.125, 0.25, 0.5 and 1.0 M GdmCl were added in A, B, C, D and E, respectively.

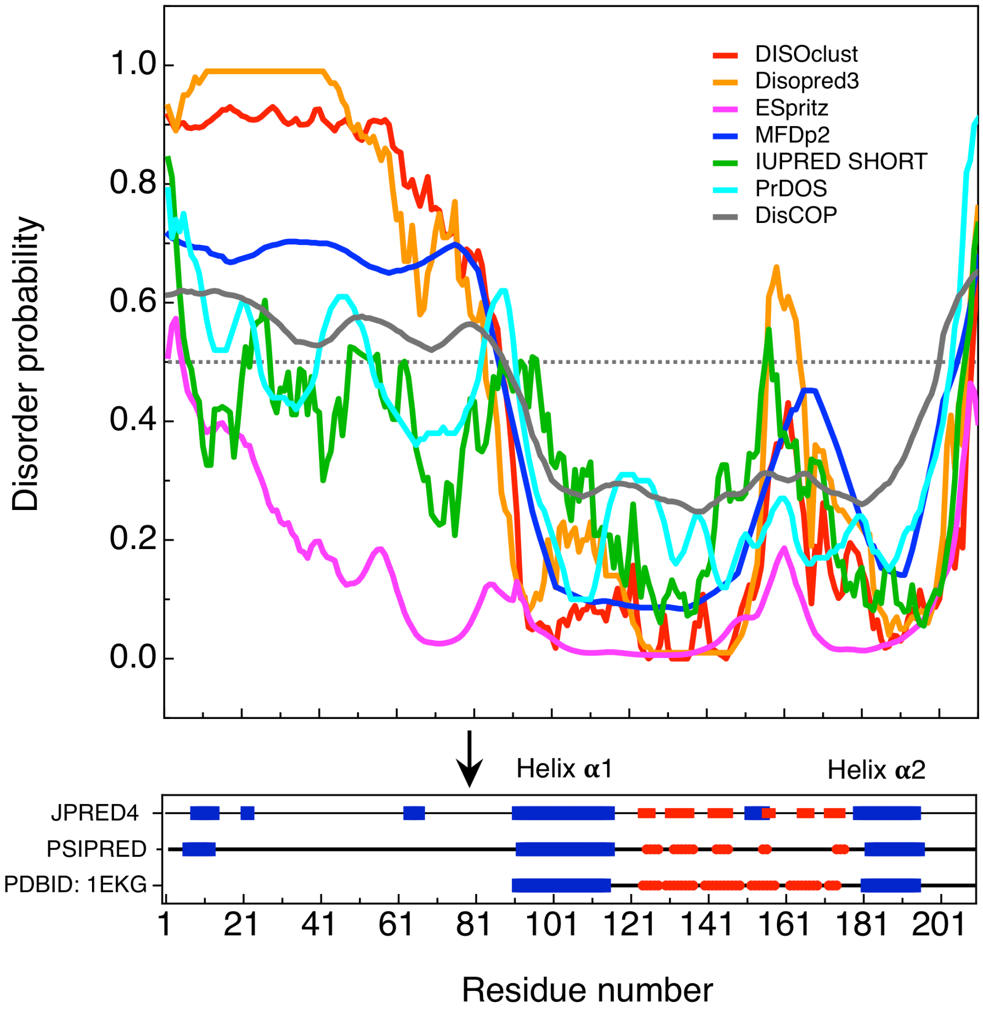

Figure S4. Predictions of Disorder in the N-terminal Segment of FXN1-210 Precursor. Disorder predictions were carried out using a number of programmes: DiSOclust (red, [1]), Disopred3 (orange, [2]), Espritz (magenta, [3]), MFDp2 (blue, [4]), IUPRED (green, [5]), PrDOS (cyan,[6]) and DisCOP (dark-gray, [7]). Disorder probability is plotted and the dotted line shows the 0.5 threshold. In the lower panel, two different predictions of secondary structure (Jpred4 [8] or PSIPRED [9]) content are plotted side by side with the secondary structure content obtained from the X-ray structure (PDB ID: 1EKG, [10]). The black arrow indicates the starting residue of the mature form of FXN (FXN81-210). Numbering corresponds to that of the precursor of FXN, for this reason His6-TAT-FXN1-210 starts at residue -19.

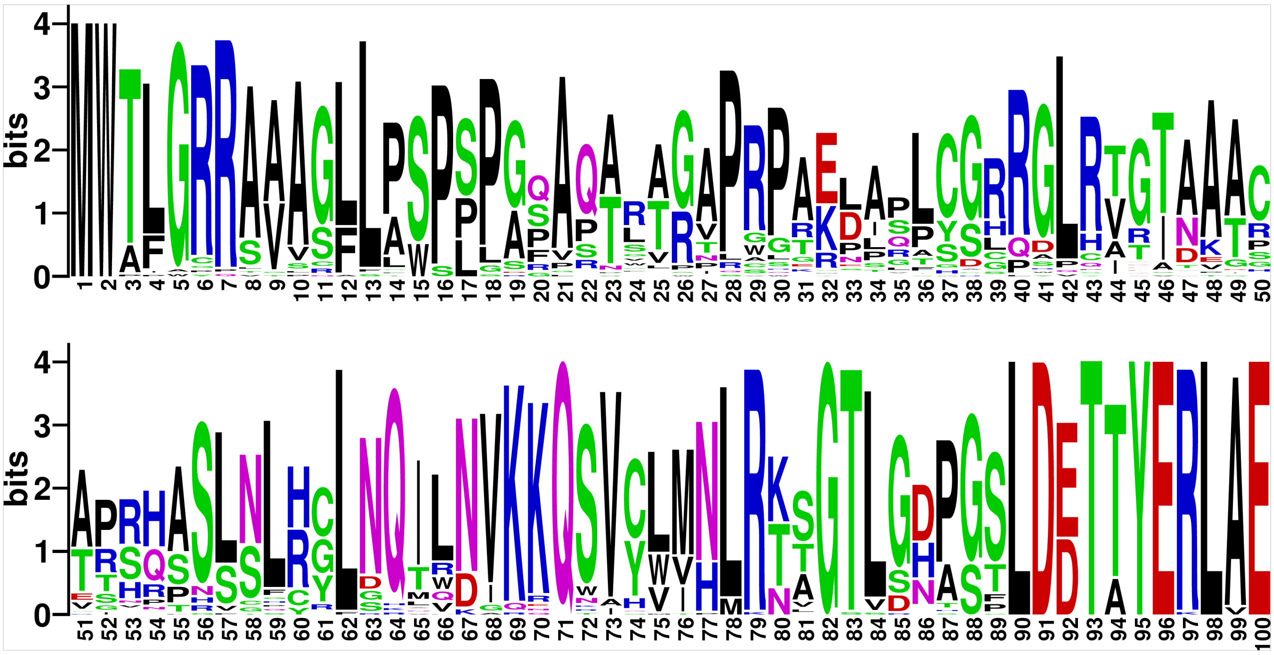

Figure S5. The N-terminal of FXN1-210 is not conserved along the evolution. LOGO corresponding to full-length human FXN (for clarity, only the 100 first residues are shown) was prepared using 50 sequences of >70% identity and >99% query covering. LOGO was created using Weblogo (http://weblogo.berkeley.edu/logo.cgi).

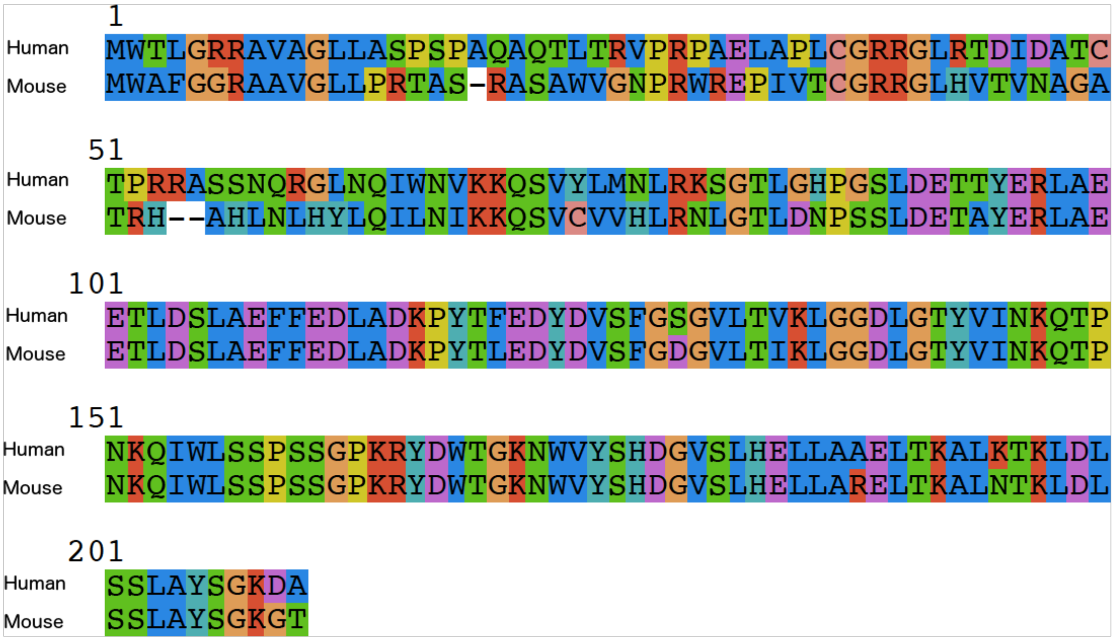

Figure S6. The N-terminal of FXN1-210 is not conserved. Sequences corresponding to the human (GenBank: AAH23633.1, up) and mouse (GenBank: AAB67778.1, down) FXN precursor are compared.

References

[1] L.J. McGuffin, Intrinsic disorder prediction from the analysis of multiple protein fold recognition models, Bioinformatics, 24 (2008) 1798-1804.

[2] D.T. Jones, D. Cozzetto, DISOPRED3: precise disordered region predictions with annotated protein-binding activity, Bioinformatics, 31 (2015) 857-863.

[3] I. Walsh, A.J. Martin, T. Di Domenico, S.C. Tosatto, M.J. Mizianty, V. Uversky, L. Kurgan, Z. Dosztanyi, V. Csizmok, P. Tompa, I. Simon, T. Ishida, K. Kinoshita, ESpritz: accurate and fast prediction of protein disorder. Bioinformatics, 28 (2012) 503-509.

[4] M.J. Mizianty, V. Uversky, L. Kurgan, Prediction of intrinsic disorder in proteins using MFDp2, Methods Mol Biol, 1137 (2014) 147-162.

[5] Z. Dosztanyi, V. Csizmok, P. Tompa, I. Simon, IUPred: web server for the prediction of intrinsically unstructured regions of proteins based on estimated energy content, Bioinformatics, 21 (2005) 3433-3434.

[6] T. Ishida, K. Kinoshita, PrDOS: prediction of disordered protein regions from amino acid sequence, Nucleic Acids Res, 35 (2007) W460-464.

[7] X. Fan, L. Kurgan, Accurate prediction of disorder in protein chains with a comprehensive and empirically designed consensus, Journal of biomolecular structure & dynamics, 32 (2014) 448-464.

[8] A. Drozdetskiy, C. Cole, J. Procter, G.J. Barton, JPred4: a protein secondary structure prediction server, Nucleic Acids Res, 43 (2015) W389-394.

[9] D.W. Buchan, F. Minneci, T.C. Nugent, K. Bryson, D.T. Jones, Scalable web services for the PSIPRED Protein Analysis Workbench, Nucleic Acids Res, 41 (2013) W349-357.

[10] S. Dhe-Paganon, R. Shigeta, Y.I. Chi, M. Ristow, S.E. Shoelson, Crystal structure of human frataxin, J Biol Chem, 275 (2000) 30753-30756.
